# Modulation of Brain Activity during Action Observation: Influence of Perspective, Transitivity and Meaningfulness

**DOI:** 10.1371/journal.pone.0024728

**Published:** 2011-09-12

**Authors:** Sébastien Hétu, Catherine Mercier, Fanny Eugène, Pierre-Emmanuel Michon, Philip L. Jackson

**Affiliations:** 1 Centre Interdisciplinaire de Recherche en Réadaptation et Intégration Sociale, Québec City, Québec, Canada; 2 École de Psychologie, Faculté des Sciences Sociales, Université Laval, Québec City, Québec, Canada; 3 Département de Réadaptation, Faculté de Médecine, Université Laval, Québec City, Québec, Canada; 4 Centre de Recherche Université Laval Robert-Giffard, Québec City, Québec, Canada; French National Centre for Scientific Research, France

## Abstract

The coupling process between observed and performed actions is thought to be performed by a fronto-parietal perception-action system including regions of the inferior frontal gyrus and the inferior parietal lobule. When investigating the influence of the movements' characteristics on this process, most research on action observation has focused on only one particular variable even though the type of movements we observe can vary on several levels. By manipulating the visual perspective, transitivity and meaningfulness of observed movements in a functional magnetic resonance imaging study we aimed at investigating how the type of movements and the visual perspective can modulate brain activity during action observation in healthy individuals. Importantly, we used an active observation task where participants had to subsequently execute or imagine the observed movements. Our results show that the fronto-parietal regions of the perception action system were mostly recruited during the observation of meaningless actions while visual perspective had little influence on the activity within the perception-action system. Simultaneous investigation of several sources of modulation during active action observation is probably an approach that could lead to a greater ecological comprehension of this important sensorimotor process.

## Introduction

Observing movements is part of our daily life. We often look at our own actions for coordination purposes, such as when we want to grab the beer offered by a vendor at a hockey game without spilling a drop. But even more frequently, we look at others making movements, such as when we watch our favourite hockey player making an incredible wrist shot. The discovery by Giacommo Rizzolatti's laboratory [1], [2], [3] of neurons in area F5 of the macaque monkey that respond both during the production of movements and when the same movements are observed (*mirror neurons*) has sparked a huge interest in the link between the processes underlying observation and execution of actions. While studies using single cell recording in non-human primates have offered convincing arguments for the presence of mirror neurons in premotor [4], [5] and parietal [6], [7] areas, the presence of neurons possessing such properties in the human homologues of these regions has yet to be confirmed [8] (but see [9] for data on human mirror neurons in other brain regions). Nevertheless, data from a large range of brain imaging techniques in humans point to a fronto-parietal perception-action coupling system, which maps actions that are observed onto the motor repertoire of the observer [10], [11], [12], [13], [14], [15], [16]. The frontal portion of this system presumably includes the inferior frontal gyrus (IFG)/ventral premotor cortex (vPM), while the parietal portion is considered to be located in the inferior parietal lobule (IPL). Note that the present paper will refer to the IFG as the frontal portion of the perception-action system but results from other studies will be discussed using either the IFG or vPM depending on the terminology used by the authors.

Most imaging studies on action observation have used a *passive* observation paradigm where participants have to observe movement without any overt aim. Passive observation of movements is part of daily life; however, often we also observe actions with the intent to reproduce them. This is true for children looking at their parents and imitating their gestures or facial expressions, but also for adults who, for example, try to learn/re-learn new movements when taking on a new sport or during rehabilitation following an injury. Up to now very few studies have looked at the functioning of the perception-action system during such *active* observation tasks (i.e., observing movements to reproduce them later). Importantly, even fewer have focused on describing how some characteristics of an observed movement can modulate the functioning of the perception-action coupling system during *active* observation. This paper is thus interested in studying the modulating effects of two of these characteristics during active action observation: the visual perspective from which the action is observed and the type of action being observed.

### How we observe: the influence of visual perspective during action observation

We observe actions from a large range of visual perspectives (VP): the poles of this continuum are the first person/egocentric VP (i.e., looking at an action made by a person facing the same direction as we are or looking at ourselves while we are moving) and the third person/allocentric VP (i.e., looking at someone's actions when they are facing us or observing ourselves moving in a mirror). The possible influence of VP on brain activity during action observation has mainly been examined by functional magnetic resonance imaging (fMRI) studies designed to compare first and third person VP. Data from the studies by Hesse and colleagues [17] and Jackson and colleagues [18] indicate that varying VP changes the brain's response during action observation, but that this modulation seems to be outside of the fronto-parietal perception-action system. Yet, results from these two studies differ on some aspects (e.g., only Hesse and colleagues found a modulating effect in the parietal lobe). Some of these differences could be explained by the fact that Hesse and colleagues used object-directed actions while Jackson and colleagues used intransitive movements (no goal or object present). This could highlight a possible interaction between VP and the type of movements observed. Importantly, studies on VP have until now only used passive observation tasks.

### What we observe: The influence of the type of movements during action observation

We are constantly exposed to a myriad of movements. For example, one night in a bar you see your girlfriend grasp a beer bottle, later on she sees you waving to the waitress to indicate that you will pick up the tab and at the end of the evening you both see the moving motion of the waitress' arms while she walks to come get your credit card. In this simple scene, several types of movements are observed: movements done with or towards objects, movements of a communicative nature and movements associated with locomotion. It is clear that these different movements vary on several aspects. Of the many aspects on which the types of movements can be differentiated, two have drawn a lot of interest from neuroscientists working on action observation: transitivity and meaningfulness.

Transitive movements include actions done with an object, such as writing with a pen, and actions done towards an object or target, such as reaching for a pen or moving a finger towards a particular key on a computer keyboard. Note that, from here on, transitivity will be defined as the presence (transitive) or absence (intransitive) of a physical object/target. Work on action observation in monkeys suggested that mirror neurons were only responsive to object-directed actions [2], [3], [19]. In humans however, transcranial magnetic stimulation (TMS) studies have shown that watching transitive [16], [20], [21], [22], [23] as well as intransitive [10], [20], [21], [24], [25], [26], [27] actions can increase the corticospinal excitability of neurons controlling the muscles involved in producing the observed movement. Increases in the corticospinal excitability within primary motor cortex (M1) during action observation measured by TMS have been shown to be mediated by regions of the perception-action system ([10], [16]see [12] for review). Several fMRI and positron emission tomography (PET) studies have also examined whether brain activity in suggested regions of the perception-action system in humans (i.e., IFG, IPL) is modulated by transitivity. Here again, data suggest that watching both transitive [17], [28], [29], [30], [31], [32], [33], [34] and intransitive [29], [30], [33], [35], [36], [37], [38], [39], [40], [41] actions recruits the perception-action system. When directly comparing transitive and intransitive movements, some authors have found increased activity during transitive actions in parietal [29] and fronto-parietal areas [42] of the perception-action system suggestive of a “preference” for object-directed actions. Others, however, have found no difference in activation between transitive and intransitive movements within the perception-action system [30], [33]. To our knowledge, the only two studies that examined the influence of transitivity using an active observation task showed conflicting results. One found that object directed movements produced higher activity in the frontal portion of the perception-action system (IFG) [43] while the other found no difference in relation to transitivity [35].

In addition to transitivity, another important characteristic of observed actions is their meaningfulness. Meaningful movements can be simple transitive movements or their pantomimes (e.g., actual pen writing or the same movement without the pen) as well as communicative gestures (e.g., salute, stop, thumbs-up). Observation of both meaningful [40], [44], [45], [46] and meaningless actions [40], [44], [45], [46], [47] has been shown to involve at least some parts of the fronto-parietal perception-action system. Nonetheless, both types of actions seem to be processed differently as at least three studies found that observation of actions can produce distinct patterns of activation for meaningful and meaningless actions [40], [44], [46]. Directly comparing activations within the perception-action system during meaningful and meaningless actions has resulted in conflicting results. While some have found increased activity during observation of meaningful movements in the left IFG [47] and left IPL [48], others have shown increased activity in the IPL during meaningless movements [45]. Importantly, these results mainly come from passive observation tasks. Considering studies that used active observation, the same conflicting pattern of results is clearly apparent as some authors have found different patterns of activity between meaningful and meaningless actions [40] while others have not observed such differences [46], [49].

Careful examination of the large body of research on action observation mentioned above reveals three main observations. First, there are conflicting results on how the type of actions we watch influences brain activity, notably within the perception-action system. Furthermore, if and how the visual perspective from which an action is observed interacts with the type of action observed remains unclear. Secondly, when investigating the influence of the type of movement during action observation, most research has focused on either transitivity or meaningfulness even though the movements we observe are often defined by more than one factor (e.g., waving to say hello is intransitive but meaningful). And finally, most studies have used passive observation tasks. Hence, the aim of the present study was to investigate how the type of movements (defined by both transitivity and meaningfulness) and the visual perspective can modulate brain activity during active action observation in healthy individuals. We hypothesised that increasing the meaningfulness, the transitivity or both during action observation would be associated with increasing levels of activity in the premotor (IFG) and parietal (IPL) regions of the perception-action system. As for the influence of the visual perspective, we expected that varying the VP would modulate brain activity but outside of the perception-action system. In order to test these hypotheses, we used an event-related fMRI paradigm in which participants had to watch transitive meaningful, intransitive meaningful and intransitive meaningless movements of the right upper-limb presented in a first person and third person visual perspective to subsequently imitate or imagine them.

## Methods

### Participants

Eighteen healthy right-handed adults (6 males) took part in this study. Participants were between 19 and 35 years old (mean = 25, SD = 4). Participants had no contraindications for MRI exams and reported no history of neurological or psychiatric disorder or musculoskeletal condition affecting their dominant upper limb.

#### Ethics statement

The study was approved by local Ethics Committees (Unité de Neuroimagerie fonctionnelle (UNF), Montréal and Institut de Réadaptation en Déficience Physique de Québec (IRDPQ), Québec City). All participants gave their written informed consent and received a financial compensation.

### Material

#### Visual Stimuli

Stimuli were two-second movie clips of a male model's right forearm and hand executing various movements on a blue background. Movements were filmed simultaneously by two cameras facing each other to produce a first person (i.e., egocentric: as if the participant was watching himself do the movement; 0 degree angle) and a third person (allocentric: as if an individual in front of the participant made the movement; 180 degree angle) visual perspective. Three types of movements were depicted in the movie clips: 1) everyday movements performed with an object (e.g., pushing a button on a pocket calculator with the index, grabbing a cup by its handle); 2) pantomimes of the same everyday movements (i.e., same gesture without the object); 3) meaningless movements made without an object (e.g., moving the hand from a pronation to a neutral position and then moving the thumb). For each condition, six different movements were filmed. A list of the meaningful movements is presented in Table 1. Movie clips were presented on a screen at the back of the MRI magnet while participants watched them through a mirror attached to the head coil. Stimulus presentation and behavioural responses were controlled and recorded using E-Prime 2 software (Psychology Software Tools, Pittsburgh, PA).

**Table 1:** Description of the stimuli.

Stimuli
1. Picking up a coffee mug by the handle
2. Pressing on a pocket calculator key with the index
3. Picking up an eraser
4. Using a television remote control
5. Using a pen (making a circular pattern)
6. Using a door key

List of the 6 different everyday movements that were presented to the participants. Note that each movement was associated with a pantomime movement where the object was absent and a meaningless hand movement. Also, each movement was presented in the first and third visual perspectives.

#### Electromyographic recording

During the scanning sessions, electromyographic (EMG) data was recorded from the right *Opponens Pollicis* (OP) and the right *Extensor Radialis Carpi Longus* (ECR) muscles. EMG recordings were obtained using a Biopac system (BIOPAC Systems Inc, Goleta, CA, USA) and translucent MRI-compatible electrodes placed in a bipolar configuration. The EMG signal was sampled at 1000 Hz, amplified (X 2000) and band-pass filtered (100–500 Hz).

### Procedure

Functional and structural scans were acquired on a 3.0 Tesla Siemens TIM Trio system with a 12 channel head coil. Before functional data acquisition, structural high-resolution T1-weighted anatomical images were acquired with a 9 m 24 s MPRAGE sequence (TR = 2300 ms, TE  = 4.94 ms, TI = 900 ms, flip angle  =  ∼25°, FOV  = 250 mm, matrix  = 256×256 voxels, voxel size  = 1×1×1 mm). During the experimental sessions, changes in blood oxygenation level-dependent (BOLD) T2- weighted signal were measured using a gradient echo-planar imaging (EPI) sequence (40×3mm contiguous axial slices parallel to the AC-PC line with 3 mm in plane resolution; TR  = 3000 ms, TE  = 30 ms, flip angle  = 90°, FOV  = 192×192 mm, 64×64 matrix). The study used an event-related design.

**Figure 1:**
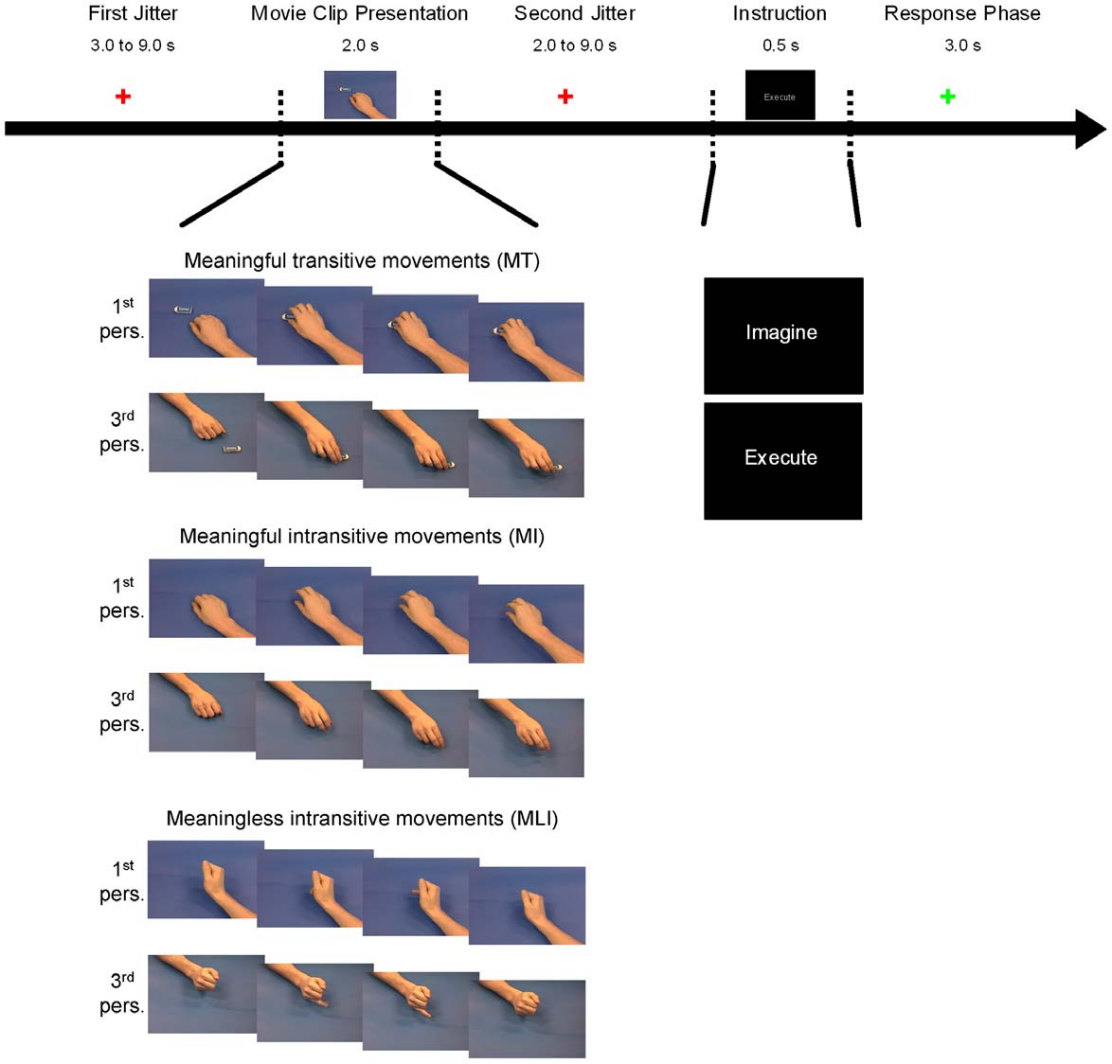
Timeline of an experimental trial. Upper section shows the five steps of a trial and their durations. Lower section shows examples of the different types of stimuli used in the experimental session. Meaningful transitive actions (MT), meaningful intransitive actions (MI) and meaningless intransitive action (MLI) were either seen in a first or third person visual perspective. The two instructions screens are also shown in the lower section.

Before entering the magnet, participants watched on a computer screen every movie clip that would later be used during the experimental sessions. This was done in an effort to increase the probability that the participants would be aware that the everyday movements done without an object were the same as the ones done with an object and thus be considered as meaningful movements and not as meaningless or aimless movements. Indeed, when questioned after the experiment, most participants mentioned being aware that some movements were the same but sometimes included an object, suggesting that this procedure was effective. After the stimuli presentation, participants were placed in the scanner and electrodes were positioned over the target muscles. During the actual task, participants had to carefully observe the various movie clips. After each movie clip presentation, participants saw a written instruction: either “Execute the movement” or “Imagine the movement”. Within a session, half the trials had the “execute” instruction while the other half had the “imagine” instruction and this was determined pseudo-randomly. Following this instruction participants either had to imitate or imagine themselves doing the movement they had just seen (response phase). Two categories of trials were shown during the experimental sessions: experimental and rest. The experimental trials started with a red fixation cross (jitter of varying durations based on a geometric distribution: 3.0, 3.5, 4.0, 4.5, 5.0, 7.0, 9.0 seconds), followed by a movie clip (2.0 s), a red fixation cross (jitter of varying durations: 2.0, 2.5, 3.0, 3.5, 5.0, 7.0, 8.0, 9.0 seconds, distributed following a geometric distribution), an instruction (0.5 s) and a response phase represented by a green fixation cross (3.0 s) (Figure 1). The rest trials consisted only of a red fixation cross lasting pseudo-randomly between 7.0 and 20.0 seconds. This BASELINE condition was included in order to obtain a condition with low levels of visual input. Three different types of movements were presented in the movie clips: 1) everyday meaningful transitive (i.e., involving an object) movements (MT); 2) the same meaningful movements but intransitive (i.e., pantomimes; MI); 3) meaningless intransitive movements (MLI). As participants saw the movements either in the first or third person visual perspective, the study comprised a total of 6 action observation conditions (MT-1, MT-3, MI-1, MI-3, MLI-1, MLI-3). Participants underwent four fMRI runs lasting approximately 10 min each. During each run, 42 trials were completed: all movie clips were pseudo-randomly presented (6 different movie clips X 6 conditions = 36 trials) in addition to six trials of the BASELINE condition. A familiarisation session of seven trials showing stimuli not used in the experimental session was done before the experimental task in the scanner to ensure that participants followed the instructions.

### Data pre-processing and analyses

#### EMG

Unsurprisingly, the EMG signal was highly affected by the magnetic field of the scanner. Therefore, the signal was first pre-processed off-line with various filters to minimize the artefacts produced by the magnets (e.g., low frequency drifts). Even after the pre-processing procedure, because of the large amount of noise produced by the magnets, the EMG data still had a low signal to noise ratio. However, it was clear that movements were associated with large changes in the amplitude/shape of the EMG signal. These important variations in the signal meant that the EMG data could be used to identify when participants moved their right upper-limb. More specifically, using the Spike2 software (Cambridge Electronic Design, Cambridge, UK), the EMG signal was thoroughly checked to identify: 1) changes in amplitude that could indicate muscle-like activity; and 2) changes in the waveform shape that could indicate muscle-like activity (e.g., frequency, general shape of the signal, etc.). Analyses of the EMG data for the two muscles were performed to screen out any trials in which the participants made any or these error types: 1) movements during the observation phase or during the BASELINE condition shown by increases/changes in EMG activity; 2) movements during the response phase following the “Imagine the movement” instruction shown by increases/changes in EMG activity; 3) no movement during the response phase following the “Execute the movement” instruction indicated by the absence of detectable increase/change in EMG activity. All trials that were identified as “error trials” were modeled as an error regressor of non-interest in the fMRI design matrix.

#### fMRI

Functional imaging processing and statistical analyses were performed with Statistical Parametric Mapping (SPM5, Wellcome Department of Cognitive Neurology, UK) software implemented in MATLAB (Mathworks Inc. Sherborn, MA, USA).

#### fMRI data pre-processing and design matrix specifications

As this study focuses on action observation, the analyses described here are mainly limited to this phase. Brain activity during the Execution trials of the response phase were only used in the conjunction analysis while data from the response phase of the Imagination trials are not reported in this paper. Each participant's imaging time series were realigned to the middle image of each run and each volume was submitted to slice timing corrections. The time series were normalized to the Montreal Neurological Institute (MNI) template and spatially normalized volumes consisted of 3×3×3 mm voxels. Finally the data were smoothed with a Gaussian kernel of 8×8×8 mm full width at half-maximum. Each Observation condition (MT-1, MT-3, MI-1, MI-3, MLI-1, MLI-3) was modeled as a regressor. Similar regressors were used for the Response phase depending on the type of observation that preceded the participant's response: (Execution: EMT-1, EMT-3, EMI-1, EMI-3, EMLI-1, EMLI-3; Imagination: IMT-1, IMT-3, IMI-1, IMI-3, IMLI-1, IMLI-3). In addition, a Baseline regressor and regressors of non-interest consisting of the error trials and head movement correction parameters were also modeled. The resulting functions were convolved to the canonical hemodynamic response function (HRF) to produce the model. Event durations were 2 seconds for the observation and Baseline events and 3 seconds for response phase events.

### fMRI analyses: Influence of visual perspective and type of movement during action observation

Experimental conditions were organized based on a 2×3 factorial design with the two factors being Visual perspective (1^st^, 3^rd^) and Type of movement (MT, MI, MLI). First, the interaction effect on BOLD activity between the Type of movement and the Visual perspective factors was verified with an *F*-contrast. Secondly, to assess if each of the factors modulated the BOLD activity, main effect analyses were performed separately for the Type of movement and VP factors (note that the main effect for Type of movement was done with an *F*-contrast while the main effect of VP was done with two *t*-contrasts: 1^st^ vs. 3^rd^ and 3^rd^ vs. 1^st^). All these analyses were done on the whole brain with a voxel by voxel analysis at the single-subject level, followed by a random effects analysis (one sample *t*-test) to investigate activations at the group level. If not explicitly mentioned, analyses were done using a family-wise error (FWE) correction with a p<0.05 and a cluster volume threshold of 10 voxels.

As we were primarily interested in studying the influence of the VP and the Type of movement on the specific regions of the perception-action system involved in action observation, we also analysed our fMRI data using a region of interest (ROI) approach. First, to define regions of the perception-action system (our ROIs), a conjunction analysis was performed to identify regions that were active during both the observation and the execution of movements. Specifically, two whole-brain analyses consisting of the contrasts OBSERVE (all conditions)-BASELINE and EXECUTE (all conditions)-BASELINE were performed. Then, using the Marsbar toolbox [50] the resulting SPM*{t}* maps were superimposed in order to identify regions that showed activation in both contrasts. Of the regions identified by this conjunction analysis, regions within the IFG or IPL were defined as ROIs since they are generally considered as being at the core of the perception-action system [11], [51]. To verify that activation loci were within the perception-action system, local maxima of clusters were labelled using Talairach daemon (after conversion from MNI to TAL). Second, using the Marsbar toolbox, ROIs were constructed as spheres of 5 mm radius centered at the center of mass of any significant activation cluster within the IFG or IPL identified at the group level by the conjunction analysis. For each ROI, parameter estimates for each action observation condition were averaged for each participant over the 4 sessions. For each ROI, possible effects of Visual perspective and Type of movement factors on the activation patterns during action observation were assessed with repeated measures (2×3) ANOVAs on the averaged parameter estimates. ROI statistical analyses were done with a level of significance set at p<0.05. Post-hoc analyses were performed with a modified step up Bonferroni procedure (Hochberg procedure [52]) aimed at adjusting the alpha value for multiple tests (presented P-values are uncorrected while α values are corrected). All statistical analyses were done using the SPSS 13 software.

To explore possible effects linked to a) the presence/absence of an object, and b) the presence/absence of meaning, post-hoc exploratory analyses using specific simple contrasts were performed at the whole brain level. More precisely, to specifically explore the influence of the presence/absence of an object in the observed movements, simple contrast analyses between the MT and MI conditions were performed. Also, the possible influence of meaningfulness of the observed movements was assessed by doing simple contrast analyses between the MI and MLI conditions. These analyses were done with a p<0.0001 (uncorrected) and a cluster volume threshold of 10 voxels.

## Results

### EMG data

One of the participants was removed from the study as his EMG analyses clearly showed that he did not comply with the task instructions. For instance, there was clear evidence of muscle contraction during the response phase of several imagination trials. Therefore, all further results are for n = 17 participants. For the remaining subjects, the average% of error trials was 4% of the Observation events, 3% of the Execute events and 4% of the Imagine events. More precisely, the following number of trials were removed from each experimental condition: MT-1: 15 (4%); MT-3: 15 (4%); MI-1:17 (4%); MI-3: 13 (3%); MLI-1: 16 (4%), MLI-3: 16 (4%).

### fMRI data

**Figure 2:**
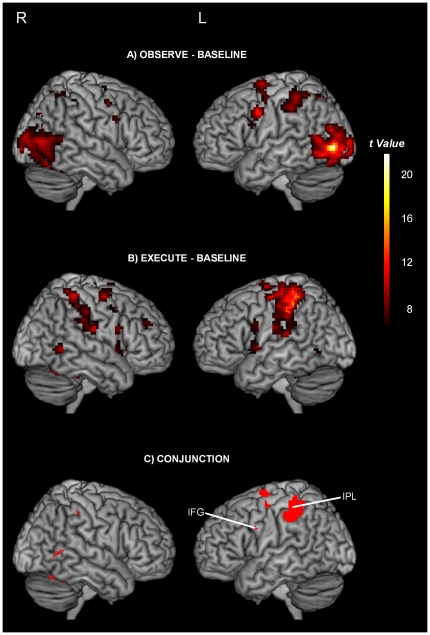
Activation sites during action observation, execution and their overlap. A) Brain regions showing a greater blood oxygenation level-dependent signal response during OBSERVE (all conditions) than during the BASELINE condition. B) Brain regions showing a greater blood oxygenation level-dependent signal response during EXECUTE (all conditions) than during the BASELINE condition. Contrast analyses are done with a FWE correction with a p<0.05 and a cluster volume threshold of 10 voxels. C) Result of the conjunction analysis of the OBSERVE-BASELINE and EXECUTE-BASELINE contrasts. Activations are superimposed on a template brain. Regions of the perception-action system are labelled. IPL: Inferior Parietal Lobule; IFG: Inferior Frontal Gyrus; R: right hemisphere; L: left hemisphere.

**Figure 3:**
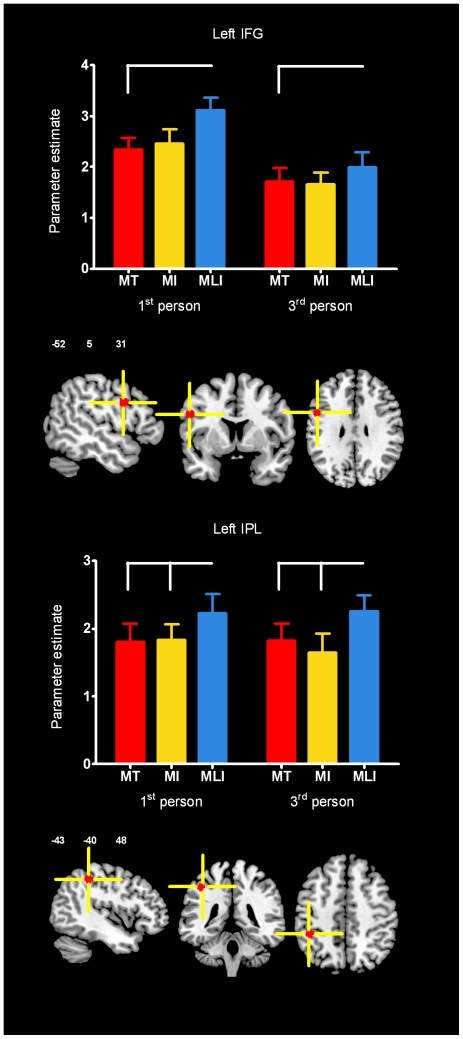
Action observation parameter estimates results for regions of the perception-action system. Results from the two regions of interest. Graphs show parameter estimates for each action observation condition: Meaningful transitive movements (MT) in red, meaningful intransitive actions (MI) in yellow and meaningless intransitive action (MLI) in blue. Activations are superimposed on a template brain with the xyz MNI coordinates of each region of interest. Error bars indicate standard error of the mean. Conditions that differ significantly are linked by white lines. IPL: Inferior Parietal Lobule; IFG: Inferior Frontal Gyrus.

Results of the whole brain analyses are shown in Figure 2 and as supporting material in Table S1. As mentioned before, we were especially interested in regions of the perception-action system located in the IFG and in the IPL. Of the activation sites identified by the conjunction between the OBSERVE-BASELINE and the EXECUTE-BASELINE contrasts (Table S2), two were located in or partially overlapped these regions: within the left IFG (MNI coordinates in mm: −52, 5, 31) and the left IPL (−43, −40, 48; Table S2 and Figure 2). Results for each ROI are shown in Figure 3.

### Interaction effect between visual perspective and type of movement on brain activity during action observation

The whole-brain analyses aimed at measuring changes in activity linked to the interaction between the Visual perspective and the Type of movement factors (*F*-contrast) revealed no significant activation site. Furthermore, focusing on the activity within the perception-action system, our ROI analyses revealed no statistically significant Visual perspective X Type of movement interaction (left IFG: *F*(2,32) = 0.986, *p* = .384); left IPL: *F*(2,32) = 0.696, *p* = .506).

These results suggest that the VP and the Type of movement factors did not interact with regards to changes in BOLD activity within or outside the perception-action system.

### Effect of the type of movements on brain activity during action observation

No main effect was found for the Type of movement factor at the whole brain level. However, our ROI analyses revealed that the main effect of the Type of movement was statistically significant in the left IFG (*F*(2,32) = 6.407, *p*<.01) and in the left IPL (*F*(2,32) = 9.394, *p*<.01). In the left IPL ROI, a post-hoc analysis revealed that MLI action observation was associated with increased activity compared to MT (*t*(33) = 3.352, *p*<.01, α = .025) and MI (*t*(33) = 5.698, *p*<.01, α = .05). Similar results were found for the IFG as the MLI condition was associated with increased activity when compared to MT (*t*(33) = 3.015, *p*<.01, α = .05) and tended to be greater when compared to MI but this difference was not significant after correction for multiple tests (*t*(33) = 2.427, *p* = .027, α = .025). There was no statistically significant difference in BOLD signal between the MT and MI conditions in either ROI. This suggests that the left IFG and left IPL were especially responsive to intransitive meaningless movements.

### Effect of visual perspective on brain activity during action observation

Our whole brain analyses (two *t*-contrasts) on the main effect of the Visual perspective factor resulted in statistically significant differences between the first and third VP in several brain regions (Table S1). The left cuneus and the middle gyrus of the occipital lobe were more active during observation of movements presented in first person than third person VP, while the right lingual gyrus of the occipital lobe was more active during observation of movements presented in third person VP when compared to first person VP. Our ROI analyses revealed no significant effect of VP on brain activity in regions of the perception-action system (left IFG: *F*(1,16) = 0.146, *p* = .707; left IPL: *F*(1,16) = 0.263, *p* = .615). These results suggest that if VP can modulate brain activity during action observation, this modulation is found outside of the regions of the perception-action system.

### Specific effect of adding an object or meaning to an action on brain activity during action observation

As the effect of the Visual perspective factor was limited to very specific regions of the occipital lobe (outside regions of the perception-action system), data for the first and third person VP were combined for the following whole brain analyses focusing on the effect of a) the transitivity and of b) the meaningfulness of the action. The simple contrast MT-MI revealed that the presence of an object was associated with increased left activations in the parahippocampal, fusiform and middle occipital gyri in addition to right activations in the fusiform and inferior occipital gyri. No significant activation was found for the opposite contrast (MI-MT). Focusing on the meaningfulness of the observed action, the simple contrast MI-MLI revealed no significant activation while the opposite contrast MLI-MI showed that observing meaningless movements, when compared to observing meaningful movements, was associated with increased bilateral activation in the IPL, and middle temporal gyrus, right hemisphere activations in the IFG and postcentral gyrus of the parietal lobe, and left hemisphere activations in the middle occipital gyrus and precuneus. Taken together, these results suggest that: 1) transitive movements activate several brain areas outside of the traditional perception-action system to a greater extent than intransitive movements; 2) intransitive meaningless movements are associated with increased activation in several brain regions including the perception-action system (IPL), compared to intransitive meaningful actions.

## Discussion

The objective of this study was to investigate the possible modulating role of the type of movements and visual perspective on the brain response during active action observation. Contrary to most previous studies in which only one characteristic was manipulated, the present work aimed at simultaneously investigating the influence of several variables that define a movement, namely transitivity, meaningfulness and visual perspective. We expected that increasing the meaningfulness, the transitivity or both during action observation would be associated with increased levels of activity in regions of the perception-action system. As for the influence of the visual perspective, it was expected to modulate activity in areas located outside the perception-action system (e.g., in the occipital lobe) and to interact with the type of movement observed. Our results show that the type of movement did modulate brain activity in the regions of the perception-action system during active action observation. Somewhat surprisingly, the main finding of this paper is that, contrary to our hypotheses, regions of the perception-action system were particularly responsive to meaningless actions made without an object. Our results also indicate that there was no interaction between the type of movement and the visual perspective on brain activity within or outside the perception-action system. Furthermore, the IFG and IPL regions -often mentioned as being the core of the perception-action system- did not respond differently to actions made with an object compared to pantomimes. These results are discussed in more details in the following sections.

### Action observation: passive vs. active tasks

One important difference between the present study and the majority of work on action observation lies in the instructions given to the participant. While in most studies participants had to passively observe movements, in the present study participants were instructed to observe and then either do a pantomime imitation or reproduce mentally the observed action. It is clear that these two different sets of instructions focus on action observation and thus should result in approximately the same cognitive process. However, when participants are asked to reproduce the movement they just observed, they have to subsequently use the information acquired during the observation, and this could result in different brain activity between active and passive action observation. Indeed, studies have already shown that distinct patterns of brain activity are associated with passive and active action observation. Notably increased activity in the IPL [53], [54] and the IFG [54] was found when participants were instructed to observe in order to subsequently execute the movements compared to being instructed to observe passively. However, a recent activation likelihood estimation (ALE) meta-analysis has shown that passive action observation was associated with more consistent activations in the IPL and IFG [55]. The meta-analysis also revealed that whereas passive action observation consistently activates the IFG and IPL, observing in order to subsequently imitate only activates the frontal part of the perception-action system. Importantly, the authors of this meta-analysis point out that only eight studies using active action observation were included in their analyses and thus that these results should be carefully considered. Indeed, our study rather suggests that similar to what is found during passive action observation tasks, both the IFG and IPL are recruited during active action observation. Taking this into account, the results of the present study will be discussed in relation to active and passive tasks as during both the core process remains the processing of visual information about human movement. Nevertheless, this important methodological difference will be considered throughout the following discussion.

### Effect of visual perspective on brain response during action observation

Our whole brain analysis on the effect of VP revealed highly lateralised responses in the occipital lobe. First person VP was associated with increased activity in the left hemisphere while third person VP activations were located in the right hemisphere. This contralateral (to the moving hand) activation in the first person VP and ipsilateral in the third person VP is consistent with results obtained by Hesse and colleagues [17]. Even if the stimuli were shot in a manner to have the moving part of the limb (the hand) in the middle of the screen for both VP conditions (see Figure 1), one cannot rule out the possibility that, taken as a whole (hand and forearm), the stimuli of each VP were differently positioned on the screen: first person VP actions mainly occupying the right side of the screen and vice versa for the third person VP. This relative lateralization of the stimuli could thus be the cause of the lateralized pattern of BOLD activity at least in the occipital cortex. Focusing on the regions of the perception-action system, none of them was modulated by VP in this study. On the contrary, in a recent paper by David and colleagues [56], a large number of regions including the IPL were mentioned as being modulated by the VP taken by the participants during action observation. David and colleagues only used the first person visual perspective (instead of first vs. third VP) but manipulated the way the participants saw the action in order to suggest that they were themselves involved (i.e., the scene was perceived as it would look from the participant's eyes) or that they were watching someone else playing (i.e., the participant saw the back of a model). Whereas our first vs. third person VP manipulation was not overtly intended to suggest self vs. other distinction (no such information was given to the participants in the instructions), the manipulation by David and colleagues was clearly aimed at suggesting this specific distinction. Taking this into account, it is possible that an explicit self vs. other distinction may modulate to a greater extent brain activity during action observation (including regions of the perception-action system) than the effect of first vs. third VP. However, as we cannot assess if our participants considered our stimuli as another person doing a movement at the first vs. third person VP or as being done by themselves vs. another individual, this interpretation remains speculative. Finally, as our results showed that Visual perspective and Type of movement did not interact in the regions of the perception-action system, they suggest that visual perspective has little effect on the perception-action system during action observation and that varying the type of movements has the same effect whether the action is seen from a first or a third person VP. However, one cannot rule out that as participants had to subsequently imitate/imagine the observed movements they may have focused on identifying the motor program needed to produce the movements rather than on the VP. This could have reduced potential differences in brain activity related to VP. As this is the first study to look at the role of VP during active action observation, more work is needed to better investigate if and how VP can modulate the action observation process when participants have to subsequently execute the movement.

### Effect of the types of movements on brain response during action observation

Several studies have focused on how brain activity during action observation is modulated by the types of movement observed. However, very few attempts have been made to simultaneously study the effect of several factors that characterize a movement. This study is to our knowledge the first to consider the type of movement as being defined by both transitivity and meaningfulness. By using three different types of movements, we were able to investigate if varying meaningfulness AND transitivity could modify the neural response to action observation. Moreover, specific differences between our three levels made it possible to focus on the specific influence of each variable. Indeed, as the meaningful transitive and meaningful intransitive movements were kinematically identical and only differed on the presence/absence of an object, we could focus on the influence of transitivity. Furthermore, as meaningful intransitive and meaningless intransitive movements only differed on presence/absence of meaning, we were also able to investigate the unique effect of meaningfulness.

### Specific influences of transitivity on brain response during action observation

Regarding the specific effect of transitivity, previous fMRI work, including two recent ALE meta-analyses [55], [57] have shown that during action observation the presence of an object produced increased activation in frontal and parietal regions of the perception-action system [29], [42] while studies using a repetition suppression paradigm have highlighted the involvement of these regions in the processing of information pertaining to objects or physical goals [58], [59], [60] and to kinematic information linked to hand-object interactions (in the vPM) [61]. However, when considering both our ROI and whole brain analyses, our data rather suggest that transitivity has little or even no modulating effect on the activity within the perception-action system. Indeed, our ROI analyses have failed to find differences between meaningful transitive and meaningful intransitive conditions in the IFG and IPL. Although our whole brain analyses revealed peaks of activity linked to the presence of an object, these activations were outside the classic fronto-parietal perception-action system (e.g., in the fusiform and middle occipital gyri and not in the IPL or IFG). Hence, our results are more in line with an early study by Koski and colleagues [30] on action observation that had found no difference in activation in frontal or parietal regions between goal directed (move towards a red dot) and intransitive actions.

Absence of a difference between brain activity during observation of transitive and intransitive meaningful movements in our study could have been linked to attentional factors. As our participants were not specifically instructed to focus on the objects present in the movie clips, it is possible that their attention was not directed towards this specific aspect of the actions. This could have reduced the effect of transitivity in our study. However, the fact that we found increased BOLD activity in the fusiform gyrus (outside the perception-action system) during transitive movements would suggest that our participants did attend to the objects presented in the stimuli. Indeed, a repetition suppression study has previously reported adaptation responses in the fusiform gyrus during observation of object manipulations possibly in relation to object identification processes [62]. However, our experimental paradigm did not allow us to confirm nor infirm the possible modulating role of attention on the differences between transitive and intransitive actions.

A more plausible explanation for these conflicting results is based on differences regarding *why* participants must observe actions. While the present study and Koski's study [30] used an active observation task, studies which have found a transitivity effect have used passive observation tasks. Hence, it is possible that regions of the perception-action system are less prone to the influence of transitivity when observation is done in order to subsequently reproduce a movement (either by imagining or imitating it) than during passive observation.

### Specific influences of meaning on brain response during action observation

Regarding the specific effect of meaningfulness, studies using active observation tasks have generated conflicting results, with some showing no effect of meaningfulness [49] while others have found that meaningless actions were associated with increased brain activity in the right IPL [40]. Our results are more in line with these last results as the simple contrast of MLI – MI conditions revealed increased activity in the IFG and IPL during the observation of meaningless actions. Because participants had to imitate/imagine the observed movements, meaningless actions may have required increased attention from participants, as they were less familiar than the other types of movements. Increased attention could thus have led to greater BOLD signal increases during the observation of meaningless movements. However, when asked after the experiment, participants did not mention that the MLI condition required more attention than the two others.

### What exactly is the modulating factor of brain activity within the perception-action system during action observation?

One important difference between previous work and the present study is that we examined simultaneously transitivity and meaningfulness to investigate the possible influence of the type of movement on brain activity during action observation. Indeed, most of the data on the mediating role of type of movement came from studies which only focused on one variable. Whereas these studies have shown that the presence/absence of objects/physical goals [30], [58], [59], [60], [61], [63] or meaning [40], [42], [44], [46], [47], [48] can modulate the response to action observation, the absence of main effect of Type of movement in our whole brain analysis would suggest that brain activity during action observation is not modulated by the type of movement observed. However, when we looked closer at the modulating effect of the type of movements within the core regions of the perception-action system (IFG and IPL) with ROI analyses we found that throughout the perception-action system (at least in the left hemisphere) BOLD activity was higher while participants watched meaningless actions than meaningful transitive or meaningful intransitive actions. Therefore, it seems that when observing different types of movements that vary on the transitivity and meaningfulness levels, it is the meaningfulness and not the presence/absence of an object that modulates the activity within the perception-action system. Furthermore, our results suggest that the perception-action system is more active, not when meaning is added, but rather when actions lack meaning.

As several studies had previously found that regions of the perception-action system responded more strongly to transitive [29], [42] and meaningful actions [47], [48], increased activity within these regions during observation of meaningless intransitive actions was somewhat surprising. However, it is important to note that these studies used passive action observation tasks whereas participants in the present study observed in order to subsequently imitate/imagine the movements. Our results using an active observation task are in line with a study by Vogt and colleagues in which participants had to practice guitar chords before being scanned while they watched and then performed either practised or unpractised (and thus unfamiliar or unknown) chords [64]. Results of this study revealed increased BOLD signal in bilateral IPL (and left vPM) during the observation of unpractised guitar chords (however, see [53] for conflicting results). Considering the similarity between our results and the ones by Vogt and colleagues which showed increased activity in the perception-action system during unfamiliar/unpractised movements, it could be interesting to consider the differences between our stimuli in terms of familiarity. Indeed, in addition to having a meaning per se, meaningful transitive and intransitive movements were also much more common to the participants than the meaningless movements and thus more familiar.

If we consider the meaningless movements in this study as being less familiar, our results could be partially explained by a novelty effect or by the fact that unfamiliar movements may have received more attention than familiar movements (odd ball effect). Both of these hypotheses could result in increased brain activity in the perception-action system during meaningless movement observation. Also, if our meaningful transitive and intransitive actions were more familiar to our participants, their repeated presentation could have resulted in participants habituating more rapidly to them, thereby diminishing the corresponding BOLD response. Furthermore, as exactly the same movements were presented in the MT and MI conditions (with and without an object) decreased brain activity for these two conditions could be due to a repetition suppression effect. However, as our paradigm was not explicitly aimed at studying the effect of attention or repetition suppression, future work is needed to measure their potential impact on our results.

Vogt and colleagues' results [64] and our own are in apparent contradiction with other studies that have shown that previous experience (and thus increased familiarity) with a movement increases the activity in the perception-action system when compared to movements with which individuals had less experience [36], [37], [65], [66]. However, when we consider the different paradigms used, whereas participants in these studies had to passively observe the movements, participants in the study by Vogt and colleagues and in the present study had to do an active action observation. Thus, diminished activity within the IPL and IFG measured during passive observation of unfamiliar movements and increased activity measured during active observation of the same type of movements indicate that the modulating role of familiarity his highly dependant on the context in which the observation takes place.

Active observation of unfamiliar/unknown movements can grossly resemble observational learning tasks where individuals have to learn to produce new movements from observing a model. Interestingly, the IPL and the IFG have been shown to be involved during observational learning [31], [54]. In line with this, in addition to the IPL, we also found increased activity in premotor regions of the perception-action system (left IFG) during meaningless compared to meaningful actions. This indicates that our meaningless movements were probably considered as unfamiliar or unknown by our participants. This also suggests that active observation of meaningless movements and observational learning may indeed involve similar processes.

During observational learning, one has to build a motor representation of the movement he observes. The less familiar the movement is, the less one can rely on simple modification of previously learned movement and the more actual building of a novel motor representation they have to do. We propose that this is similar to what happened during our active observation tasks. The fact that in our study the movements had to be reproduced after being watched implies that, in order to imitate the model's movements, participants had to access the precise set of motor commands necessary to activate the correct muscular groups associated with each movement. Meaningful actions presented in the present study were everyday movements that are frequently used and seen (e.g., using a pen, grasping a coffee mug). Consequently, our meaningful movements were probably more easily associated with motor representations already present in the participants' motor repertoire than our meaningless movements which were much less familiar. Whereas the motor representations/commands of familiar movements are probably accessed during action observation through motor resonance processes (i.e., observed actions being mapped onto the motor repertoire of the individual), unknown or unfamiliar actions probably have incomplete representations and thus resonance responses are likely to be less effective.

### Observing meaningless intransitive movements: is the perception-action system learning?

A growing number of authors now consider resonance responses as being a product of a special case of perception-action associative learning [8], [67], [68], [69]. Supporters of this view consider that mirror neuron or motor resonance responses are obtained after the combination of observed and executed actions at the neuronal level through a hebbian-like process taking place in the perception-action coupling system. Hence, increased activity in the perception-action system may arise from pure resonance responses: activity of motor neurons that have “learned” to be active during the observation of movements they code for. Still, our results lead us to further suggest that during observation of movements that have to be reproduced, some activity within the perception-action system may also be the product of an ongoing process attempting to link observed actions to already present motor representations. In the particular case of unknown or unfamiliar movements, increased activity (when compared to well known movements) could be linked to a need for greater involvement of the perception-action system. This increased activity could be necessary to create new associations between sensory and motor representations. Importantly, the present paradigm did not allow us to directly test this hypothesis; future research should therefore try to verify if and how mirror neurons are formed during the observation of new/unknown movements that need to be executed.

### Conclusion

By manipulating the visual perspective, transitivity and meaningfulness of observed movements to be subsequently imitated or imagined, we were able to show that the fronto-parietal regions of the perception-action system are mostly recruited during the observation of actions outside of an individual's motor repertoire. Simultaneous investigation of multiple sources of modulation during action observation is probably an approach that is bound to offer a more global and ecological comprehension of this important sensorimotor process.

## Supporting Information

Table S1 **Results from contrast analyses.** List and coordinates of the regions showing greater blood oxygenation level-dependent signal response obtained from the contrast analyses. Contrast 1 identified regions activated during action observation and contrast 2 regions activated during action execution. The effect of visual perspective was investigated with contrasts 3 and 4. The specific influence of the presence/absence of objects was investigated with contrasts 5 and 6. The specific influence of the presence/absence of meaning was investigated with contrasts 7 and 8. Coordinates are in Montreal Neurological Institute (MNI) stereotaxic space. BA: Approximate Brodmann's area; 1: first person visual perspective; 3: third person visual perspective; MT: Meaningful Transitive movements; MI: Meaningful Intransitive movements; MLI: Meaningless Intransitive movements Contrasts 1 to 4 are done using a FWE correction with a p<0.05 and a cluster volume threshold of 10 voxels. Contrasts 5 to 8 are done using a p<0.0001 (uncorrected) and a cluster volume threshold of 10 voxels.
(DOC)

Table S2 **Coordinates of the areas resulting from the conjunction analysis.** List and coordinates of the areas that showed overlapping activity for the OBSERVE-BASELINE and EXECUTE-BASELINE contrasts. Coordinates are in Montreal Neurological Institute (MNI) stereotaxic space.
(DOC)
