# Supplementary material for: Modulation of Brain Activity during Action Observation: Influence of Perspective, Transitivity and Meaningfulness
Source: PLoS One. 2011 Sep 12;6(9):e24728. doi: 10.1371/journal.pone.0024728 (PMC3171468; doi:10.1371/journal.pone.0024728)
Supplement: Table S1 — Results from contrast analyses. List and coordinates of the regions showing greater blood oxygenation level-dependent signal response obtained from the contrast analyses. Contrast 1 identified regions activated during action observation and contrast 2 regions activated during action execution. The effect of visual perspective was investigated with contrasts 3 and 4. The specific influence of the presence/absence of objects was investigated with contrasts 5 and 6. The specific influence of the presence/absence of meaning was investigated with contrasts 7 and 8. Coordinates are in Montreal Neurological Institute (MNI) stereotaxic space. BA: Approximate Brodmann's area; 1: first person visual perspective; 3: third person visual perspective; MT: Meaningful Transitive movements; MI: Meaningful Intransitive movements; MLI: Meaningless Intransitive movements Contrasts 1 to 4 are done using a FWE correction with a p<0.05 and a cluster volume threshold of 10 voxels. Contrasts 5 to 8 are done using a p<0.0001 (uncorrected) and a cluster volume threshold of 10 voxels. (DOC) [file pone.0024728.s001.doc]

|  |  |  |  | **Coordinates** | | |
| --- | --- | --- | --- | --- | --- | --- |
|  | **Brain area** | **BA** | ***t* Value** | ***x*** | ***y*** | ***z*** |
|  |  |  |  |  |  |  |
| **1. Observe vs. Baseline** | Left inferior parietal lobule | 40 | 11.54 | -39 | -39 | 45 |
|  | Left inferior parietal lobule | 40 | 10.59 | -45 | -39 | 54 |
|  | Left lingual gyrus of the occipital lobe | 17 | 14.19 | -12 | -96 | -3 |
|  | Left medial frontal gyrus | 6 | 11.03 | -9 | -3 | 60 |
|  | Left middle frontal gyrus | 6 | 10.02 | -21 | -12 | 54 |
|  | Left middle occipital gyrus | 19 | 22.51 | -39 | -81 | 0 |
|  | Left precuneus of the parietal lobe | 7 | 8.32 | -24 | -72 | 33 |
|  | Left precuneus of the parietal lobe | 7 | 13.30 | -27 | -51 | 42 |
|  | Left precuneus of the parietal lobe | 7 | 7.85 | -12 | -75 | 45 |
|  | Left precentral gyrus of the frontal lobe | 6 | 13.41 | -51 | 3 | 39 |
|  | Left subgyral of the frontal lobe | 6 | 10.98 | -18 | 0 | 59 |
|  | Right inferior frontal gyrus | 9 | 8.98 | 63 | 9 | 27 |
|  | Right middle frontal gyrus | 6 | 9.94 | 42 | 0 | 48 |
|  | Right postcentral gyrus of the parietal lobe | 2 | 10.50 | 39 | -30 | 42 |
|  | Right precuneus of the parietal lobe | 7 | 12.75 | 24 | -54 | 51 |
|  | Right lingual gyrus of the occipital lobe | 17 | 13.55 | 15 | -93 | -3 |
| **2. Execute vs. Baseline** | Left postcentral gyrus of the parietal lobe | 2 | 22.04 | -45 | -30 | 39 |
|  | Left precentral gyrus of the frontal lobe | 4 | 17.66 | -30 | -27 | 57 |
|  | Left precentral gyrus of the frontal lobe | 4 | 17.59 | -36 | -21 | 54 |
|  | Left inferior frontal gyrus | 44 | 8.77 | -54 | 3 | 24 |
|  | Left superior temporal gyrus | 22 | 10.86 | -48 | 0 | 3 |
|  | Left middle frontal gyrus | 9 | 8.42 | -33 | 30 | 30 |
|  | Left middle frontal gyrus | 10 | 8.39 | -30 | 39 | 27 |
|  | Left precuneus of the parietal lobe | 7 | 9.67 | -12 | -72 | 48 |
|  | Left insula | 13 | 10.02 | -39 | 0 | 9 |
|  | Left thalamus |  | 11.94 | -18 | -24 | 9 |
|  | Left culmen of the anterior lobe |  | 13.04 | -30 | -54 | -27 |
|  | Right inferior parietal lobule | 40 | 13.71 | 42 | -39 | 51 |
|  | Right middle frontal gyrus | 6 | 13.04 | 33 | -3 | 57 |
|  | Right inferior frontal gyrus | 45 | 9.95 | 57 | 12 | 21 |
|  | Right postcentral gyrus of the parietal lobe | 3 | 12.29 | 63 | -15 | 24 |
|  | Right postcentral gyrus of the parietal lobe | 2 | 11.62 | 48 | -21 | 30 |
|  | Right middle temporal gyrus | 22 | 11.07 | 54 | -51 | 0 |
|  | Right superior temporal gyrus | 22 | 8.76 | 51 | 9 | 0 |
|  | Right middle frontal gyrus | 10 | 8.74 | 36 | 42 | 30 |
|  | Right insula | 13 | 12.55 | 42 | 3 | 9 |
|  | Right insula |  | 9.09 | 33 | 21 | 3 |
|  | Right thalamus |  | 8.43 | 12 | -3 | 6 |
|  | Right lentiform nucleus |  | 8.27 | 18 | -3 | -3 |
|  | Right thalamus |  | 8.18 | 18 | -9 | 9 |
|  | Right culmen of the anterior lobe |  | 16.67 | 33 | -54 | -27 |
|  | Right culmen of the anterior lobe |  | 16.62 | 27 | -35 | -30 |
|  | Right culmen of the anterior lobe |  | 10.40 | 6 | -63 | -15 |
|  |  |  |  |  |  |  |
| **3. Perspective: 1 vs. 3** | Left cuneus | 17 | 9.89 | -9 | -96 | 3 |
|  | Let middle occipital gyrus | 18 | 8.47 | -18 | -93 | 15 |
|  |  |  |  |  |  |  |
| **4. Perspective: 3 vs. 1** | Right lingual gyrus of the occipital lobe | 18 | 12.53 | 12 | -84 | -6 |
|  | Right lingual gyrus of the occipital lobe | 18 | 11.77 | 24 | -81 | -12 |
|  |  |  |  |  |  |  |
| **5. Transitivity: MT vs. MI** | Left lingual gyrus of the occipital lobe | 18 | 8.15 | -30 | -72 | -15 |
|  | Left middle occipital gyrus | 18 | 7.45 | -27 | -81 | -12 |
|  | Left parahippocampal gyrus of the limbic lobe | 19 | 7.23 | -30 | -55 | -9 |
|  | Right fusiform gyrus of the occipital lobe | 19 | 7.98 | 30 | -60 | -12 |
|  | Right fusiform gyrus of the temporal lobe | 19 | 5.30 | 30 | -42 | 18 |
|  | Right inferior occipital gyrus | 19 | 5.50 | 33 | -78 | -12 |
|  |  |  |  |  |  |  |
| **6. Transitivity: MI vs. MT** | No suprathreshold site |  |  |  |  |  |
|  |  |  |  |  |  |  |
| **7. Meaning: MI vs. MLI** | No suprathreshold site |  |  |  |  |  |
|  |  |  |  |  |  |  |
| **8. Meaning: MLI vs. MI** | Left inferior parietal lobule | 40 | 11.77 | -48 | -33 | 45 |
|  | Left inferior parietal lobule | 40 | 7.23 | -39 | -35 | 45 |
|  | Left inferior parietal lobule | 40 | 5.82 | -33 | -42 | 42 |
|  | Left middle temporal gyrus | 37 | 6.52 | -51 | -66 | 3 |
|  | Left precuneus of the occipital lobe | 31 | 5.69 | -15 | -57 | 51 |
|  | Left middle occipital gyrus | 19 | 5.22 | -39 | -75 | 3 |
|  | Right inferior parietal lobule | 40 | 8.20 | 35 | -45 | 53 |
|  | Right inferior parietal lobule | 40 | 5.89 | 39 | -39 | 45 |
|  | Right inferior frontal gyrus | 9 | 6.87 | 57 | 9 | 24 |
|  | Right middle temporal gyrus | 37 | 6.44 | 48 | -63 | -3 |
|  | Right postcentral gyrus of the parietal lobe | 2 | 10.43 | 57 | -21 | 30 |
|  |  |  |  |  |  |  |
