# Supplementary material for: Modulation of Brain Activity during Action Observation: Influence of Perspective, Transitivity and Meaningfulness
Source: PLoS One. 2011 Sep 12;6(9):e24728. doi: 10.1371/journal.pone.0024728 (PMC3171468; doi:10.1371/journal.pone.0024728)
Supplement: Table S2 — Coordinates of the areas resulting from the conjunction analysis. List and coordinates of the areas that showed overlapping activity for the OBSERVE-BASELINE and EXECUTE-BASELINE contrasts. Coordinates are in Montreal Neurological Institute (MNI) stereotaxic space. (DOC) [file pone.0024728.s002.doc]

| **Brain area** | **x** | **y** | **z** |
| --- | --- | --- | --- |
| Left inferior frontal gyrus | -52 | 5 | 31 |
| Left inferior parietal lobule | -43 | -40 | 48 |
| Right posterior lobe of the cerebellum | 33 | -55 | -22 |
| Right postcentral gyrus | 40 | -32 | 44 |
| Right middle temporal gyrus | -54 | -56 | 4 |
| Left subgyral of the frontal lobe | -17 | -4 | 59 |
